# Reaction Engineering of In Vitro Natural Product Biosynthesis: Challenges and Strategies

**DOI:** 10.1002/cbic.202500571

**Published:** 2025-10-06

**Authors:** Elsa Sánchez‐García, Stephan Lütz, Markus Nett

**Affiliations:** ^1^ Department of Biochemical and Chemical Engineering Chair of Computational Bioengineering TU Dortmund University Emil‐Figge‐Str. 66 44227 Dortmund Germany; ^2^ Department of Biochemical and Chemical Engineering Chair of Bioprocess Engineering TU Dortmund University Emil‐Figge‐Str. 66 44227 Dortmund Germany; ^3^ Department of Biochemical and Chemical Engineering Laboratory of Technical Biology TU Dortmund University Emil‐Figge‐Str. 66 44227 Dortmund Germany

**Keywords:** biomolecular simulation, biosynthesis, enzyme cascade, natural product, reaction engineering

## Abstract

Natural products are widely used as pharmaceuticals and agrochemicals, or as active ingredients in food and cosmetics. Their biosynthesis typically involves a series of enzyme‐controlled reactions in dedicated liquid environments. The reconstruction of these multistep transformations under in vitro conditions bears significant potential for technical utilization. However, the concurrent operation of multiple enzymes in a single reaction flask or reactor is often associated with major challenges. Herein, the difficulties in reaching high substrate conversion and product yields with in vitro enzyme cascades are summarized. Furthermore, both established and emerging concepts for improving their performance are discussed.

## Graphical abstract

The yield and turnover of in vitro natural product biosynthesis, and even its feasibility, are determined by intermolecular interactions. Reaction engineering allows to improve the performance of such complex biocatalytic systems.

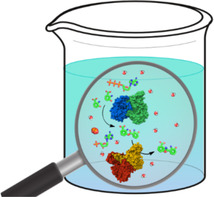

## 1 Introduction

Nature is capable of assembling complex molecules from a limited set of structurally simple building blocks. Its biosynthetic proficiency is most impressively demonstrated by secondary metabolism, which delivers a variety of bioactive natural products ranging from amino acid‐derived compounds (alkaloids, peptides, and phenylpropanoids) to polyketides and isoprenoids. The necessary chemical transformations leading to these secondary metabolites are usually carried out by proteins with catalytic activity (i.e., enzymes). Unlike their counterparts in chemocatalysis, most enzymes prefer ambient conditions and operate in a highly chemo‐, regio‐, and stereoselective fashion. Another prevalent feature of these biocatalysts is that they act in a concerted manner. Enzymes constitute metabolic pathways, in which the product of one conversion becomes the substrate of the following biocatalytic reaction. Noteworthy, enzyme‐driven reaction cascades are not always organized in a sequential manner. According to the timeline of the catalytic steps, it is possible to distinguish linear, parallel, orthogonal, and cyclic cascades.^[^ ^1^ ^]^ In any case, metabolic pathways prevent an excessive accumulation of reaction intermediates and achieve a full conversion of the substrate due to equilibria displacement.^[^ ^1^ ^]^ The maintenance of low reactant concentrations promotes high selectivity. It reduces the formation of side products and helps to avoid stability‐ or toxicity‐related issues of reactive intermediates.^[^ ^1^ ^]^

Due to these characteristics, enzymatic reaction cascades attract particular attention from chemists and process engineers alike.^[^ ^1^ ^,^ ^2^ ^]^ Especially the stereoselectivity of enzyme‐catalyzed reactions in combination with the possibility to avoid purification steps of reaction intermediates bears great potential for lowering operating time, costs, and waste in multi‐step syntheses, while atom economy and yields can be improved.^[^ ^3^ ^]^ Up to now, however, the use of enzyme cascades outside cells has been confronted with a number of challenges.^[^ ^4^ ^]^

In this review, we will delve into the field of enzymatic cascades. Our focus will be on the reconstruction of natural product biosynthetic pathways with isolated enzymes. Artificial enzyme cascades will be addressed only as far as the corresponding work provides new insights for improving the cascade's performance. For more information on the cell‐free expression of natural product pathways using cell extracts, the reader is referred to the excellent review by Rice et al.^[^ ^5^ ^]^ We will discuss the various challenges in achieving high product yields under in vitro conditions with purified enzymes, as well as the existing strategies to overcome them. Our coverage is not meant to be comprehensive but rather to illustrate existing optimization approaches for biosynthetic cascades and their enzymes, ranging from reaction engineering to biomolecular simulations.

## 2 Total In Vitro Biosynthesis

The full reconstruction of secondary metabolite pathways with purified enzymes started about 25 years ago.^[^ ^6^, ^7^ ^–^ ^8^ ^]^ Originally driven by the motivation to unravel biosynthetic reaction sequences and to better understand the interactions of the involved enzymes, it has found further applications since then, e.g., in pathway engineering and the creation of new biomolecules by combinatorial biosynthesis.^[^ ^9^ ^]^ The in vitro biosynthesis of natural product analogs has, in fact, clear advantages in comparison to fermentation‐based approaches, as it avoids cellular uptake and toxicity issues of (nonnative) substrates as well as cross‐reactivity with other metabolic pathways.^[^ ^10^ ^]^

**Figure 1:**
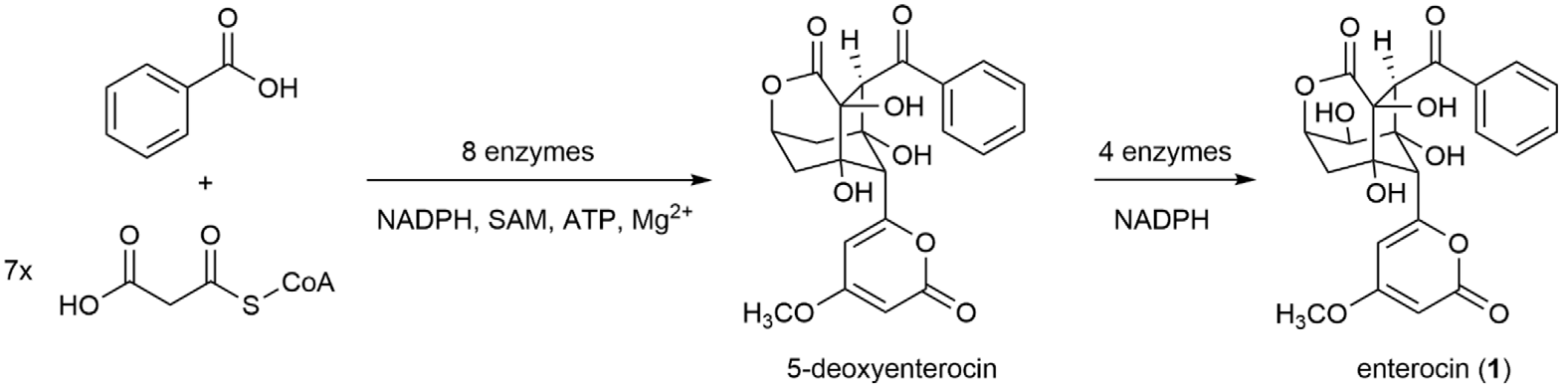
Enzymatic in vitro biosynthesis of enterocin (**1**) from benzoic acid and malonyl‐CoA. The reaction cascade involved an intermediary extraction step, since one late pathway enzyme is inhibited by SAM.^[^ ^11^ ^]^

An early study illustrating the tremendous potential of total in vitro biosynthesis was the generation of the structurally complex polyketide enterocin (**1**).^[^ ^11^ ^]^ For this, the pathway to this natural product was reconstituted with nine recombinant and three commercial enzymes. Starting from benzoic acid and malonyl‐CoA, a total of ten C—C bonds, five C—O bonds and seven stereogenic centers were formed in a two‐step cascade involving the intermediary extraction of 5‐deoxyenterocin (**Figure** **1**). Later, it was demonstrated that the in vitro system also allows the production of novel enterocin variants.^[^ ^12^ ^]^ Noteworthy, the first chemical total synthesis of **1** with its highly oxygenated tricyclic core structure was not accomplished until 2021. It involves 22 steps and achieves an overall yield of 0.4%.^[^ ^13^ ^]^

### 2.1 Challenges

Up to now, the in vitro reconstruction of secondary metabolite pathways continues to stimulate biosynthesis research.^[^ ^14^ ^,^ ^15^ ^]^ Surprisingly, however, the product yields of in vitro cascades are often much lower than those reached under cellular conditions. An analysis of literature data reveals that with few exceptions, such as amorpha‐4,11‐diene (**2**),^[^ ^16^ ^]^ only a minor fraction of the substrate is converted into the desired product (**Table** **1**).

**Table 1:** Examples of natural products whose pathways were fully reconstituted in vitro with isolated enzymes, including the required substrates and cofactors, as well as the yield of the end product. Please note that the aim of most referenced studies was the elucidation of biosynthetic reaction sequences rather than achieving an efficient bioprocess. Abbreviations: SNAc, *N*‐acetylcysteamine thioester; DMAPP, dimethylallyl pyrophosphate; GPP, geranyl pyrophosphate; n.r., not reported.

Natural product	Number of enzymes	Substrates	Cosubstrates/cofactors	Reaction medium	Optimization involved	Product yield [%]	Ref.
Pinocembrin	2	Cinnamoyl‐SNAc, malonate	ATP, MgCl_2_, coenzyme A	Tris‐HCl buffer	Enzyme immobilization	10	[28]
Naringenin	3	4‐coumarate, malonyl‐CoA	ATP, MgCl_2_, coenzyme A	Unspecified buffer	Enzyme selection, reactant and enzyme concentration	2	[138]
Camalexin	3	l‐tryptophan, l‐cysteine	NADPH	Phosphate buffer	–	1	[139]
Psilocybin	3	4‐hydroxy‐l‐tryptophan	ATP, MgCl_2_, SAM	Phosphate buffer	–	26	[62]
Dihydro‐monacolin L	3	Malonate	ATP, MgCl_2_, coenzyme A, SAM	Phosphate buffer	–	3	[140]
Merochlorin A	4	DMAPP, GPP, malonyl‐CoA	MgCl_2_, Na_3_VO_4_	MOPS buffer	–	n.r.	[141]
Ikarugamycin	4	Acetyl‐CoA, malonyl‐CoA, l‐ornithine	ATP, MgCl_2_, NADPH, FAD	HEPES buffer	–	9	[142]
Raspberry ketone	5	l‐tyrosine, malonate	ATP, MgCl_2_, NADPH, coenzyme A	Phosphate buffer	Enzyme selection	34	[143]
Cystargolide B	5	3‐isopropyl‐malate, l‐valine	ATP, MgCl_2_, SAM	Phosphate buffer	–	64	[14]
Amorpha‐4,11‐diene (**2**)	6	Mevalonate	ATP, MgCl_2_	Tris‐HCl buffer	Enzyme selection, enzyme concentration, buffer composition, product removal	100	[16]
Rabelomycin	7	Acetyl‐CoA, malonyl‐CoA	NADPH, MgCl_2_	Phosphate buffer	–	2	[144]
6‐Deoxy‐erythronolide B (**4**)	7	Propionyl‐CoA, methyl‐malonate	ATP, MgCl_2_, NADPH, coenzyme A	Phosphate buffer	–	28	[145]
Farnesene	9	Acetyl‐CoA	ATP, MgCl_2_, NADPH	Phosphate buffer	Enzyme selection, reactant and enzyme concentration	34	[146]
Patchoulol	11	Acetate	ATP, MgCl_2_, NADPH, coenzyme A	MVA buffer	Enzyme selection, enzyme concentration, cascade operation mode, product removal	40	[40]
Enterocin (**1**)	12	Benzoate, malonyl‐CoA	ATP, MgCl_2_, SAM	Tris‐HCl buffer	Cascade operation mode	25	[11]
Defuco‐gilvocarcin M	15	Acetyl‐CoA, malonyl‐CoA	FAD, MgCl_2_, NADPH, SAM	Phosphate buffer	–	20	[147]

While this is by itself not detrimental to fundamental research, low yields affect the preparation of larger product quantities and, thereby, the technical use of biosynthetic cascades. Before discussing possible strategies to overcome this issue, we will take a look at the reasons for the low product yields under in vitro conditions. Various hypotheses might explain the discrepancy in the performance of biosynthetic cascades under in vivo and in vitro conditions.

From a thermodynamics perspective, it becomes evident that reaction equilibria prevent a full substrate conversion in closed in vitro environments. Living cells are thermodynamically open systems. The product of an enzymatic reaction is continuously withdrawn, be it by subsequent conversion, by diversion into another pathway, or by cellular export. In this way, cells maintain the metabolic flux and prevent an enzymatic cascade from reaching chemical equilibrium. Another apparent difference between in vivo and in vitro systems is the composition of the reaction environment. As opposed to the dilute buffer solutions commonly used for biochemical studies, the intracellular environment is highly heterogeneous and viscous. Furthermore, it is crowded with diverse chemical constituents. The manifold chemical and electrostatic interactions occurring in the cytosol result in non‐ideal solution behavior. This has important consequences not only for enzyme kinetics, but also for the driving force of the catalyzed reaction (i.e., the Gibbs energy of reaction Δ^R^ *G*). For instance, it was shown that the feasibility of glycolysis depends substantially on the molecular interactions of salts and crowding agents.^[^ ^17^ ^,^ ^18^ ^]^ Although further experiments are necessary to validate the generalizability of this finding, such influences could explain certain regulatory effects within cells to synchronize enzyme‐catalyzed reactions.

The balancing of activity ratios and reaction conditions for the different biocatalysts within a cascade poses indeed a considerable challenge for in vitro biosynthesis. Enzymes originating from a single organism, especially those belonging to the same pathway, are usually supposed to have similar reaction optima. While this assumption is often valid with regard to pH and temperature preferences, it neglects specific requirements concerning cofactors and oxygen concentration, as well as potential crosstalk with other constituents of the cascade. To address these individual needs, living cells create dedicated microenvironments for enzymatic reactions.^[^ ^19^ ^]^ Such microenvironments can be extremely durable when confined by membranes, as exemplified by the peroxisomes and lysosomes of eukaryotic cells. In other cases, however, cells resort to dynamic compartmentalization.^[^ ^20^ ^]^ Crowding and phase separation effects cause the formation of biomolecular condensates, which help to adjust the local enzyme concentration and the diffusion of substrates.^[^ ^20^, ^21^ ^–^ ^22^ ^]^ Compartmentalization enables the tailoring of the individual reaction conditions to the biocatalyst. By segregating biosynthetic reactions, cells can avoid incompatibilities between reactants and enzymes that would otherwise affect a cascade`s performance. An example is the inhibitory effect of the cofactor *S*‐adenosyl‐l‐methionine (SAM) on cytochrome P450 enzymes.^[^ ^23^ ^]^ This incompatibility was observed in the reconstitution of enterocin biosynthesis and motivated the two‐step cascade setup displayed in Figure 1.^[^ ^11^ ^]^

Another possible barrier to high yields can be the limited operational stability of an enzyme under in vitro conditions. The preservation of an enzyme's biological structure and, thus, its activity arises from different intramolecular forces and interactions with the surrounding solvent. Again, we must allude to the fundamental differences between the cytosol and the reaction media used in pathway reconstitutions. Last but not least, one should not forget the importance of physiological concentrations for the quantitative conversion of a substrate in a biosynthetic cascade. Many enzymes are subject to allosteric regulation. Excessive concentrations of substrates or coenzymes, as well as the accumulation of products, can hence lead to their inhibition. Spontaneous, non‐enzymatic side reactions and the triggering of catabolic pathways also become more likely.^[^ ^24^ ^]^

### 2.2 Reaction Engineering Strategies

There is ample evidence in the literature showing how process adjustments can circumvent at least some of the aforementioned difficulties and even be beneficial for an enzymatic reaction. In general, reaction engineering comprises adjusting the reaction phase or the reactor setup. While the reaction phase allows for many modifications, finding an overlapping process window for multiple enzymes in terms of pH, temperature, and concentrations of reactants (substrates as well as cofactors) is challenging in cascade design. This means that typically, not all of the enzymes in an in vitro cascade will be utilized to their maximum potential in terms of productivity. In terms of reactor setup, batch reactors are typically preferred for cascades, as the enzyme ratios can be easily adjusted. It is noteworthy that in reaction engineering, the term reactor does not necessarily relate to a macroscopic technical vessel, but rather the mode of operation. As long as all the substrates and reactants are dosed into a reaction vessel at the start, the reaction is considered to be carried out in batch mode. This is important, as some of the strategies used to improve established biocatalytic reactions on technical or laboratory scale, like modification of the liquid phase with cosolvents, can also be transferred to in vitro biosynthesis cascades on a small scale.

An alternative reactor setup is the plug‐flow reactor (PFR), which requires a packed bed of enzymes immobilized on a solid support. While enzyme immobilization can enable very productive continuously operated flow reactors,^[^ ^25^ ^]^ these reactors typically only contain one reaction step and have not been applied to reconstitute a full biosynthetic pathway. Most of these reactors aim at producing pharmaceutical building blocks, while recently, the natural product pseudochelin A was also obtained in a flow reactor.^[^ ^26^ ^]^ In addition to enabling different reactor setups and compartmentalization,^[^ ^27^ ^]^ immobilization can also increase enzyme stability. The Dordick group used a rather simple procedure to immobilize chalcone synthase (CHS) and malonyl‐CoA synthetase (MCS).^[^ ^28^ ^]^ The two enzymes were expressed as fusion proteins either with a histidine or a glutathione S‐transferase tag, thus allowing their attachment onto Ni^2+^‐nitrilotriacetic acid agarose beads and glutathione‐sepharose, respectively. Subsequent testing revealed that, especially, the in vitro stability of CHS was greatly enhanced following this treatment. After immobilization, the coupled CHS/MCS reaction system achieved a 30‐fold higher yield of pyrone products compared to the free enzyme solution.^[^ ^28^ ^]^

Most of the reaction engineering approaches, however, focus on the reaction medium itself and on creating a favorable liquid environment for the enzymes and the respective reactants in batch mode. In technical biocatalytic applications, substrates are used up to molar concentrations, where solubility can become limiting. Therefore, several approaches have been developed to address this issue (**Table** **2**).^[^ ^29^, ^30^, ^31^ ^–^ ^32^ ^]^

**Table 2:** Examples of liquid reaction systems and modifications for in vitro biocatalysis.

Homogeneous systems
Aqueous buffer with water‐miscible organic solvent or ionic liquids
Micro‐aqueous reaction systems (MARS)
Neat substrate/pure solvent
Additives (e.g., sugars, crowding agents, salts)
Supercritical fluids
Water‐miscible ionic liquids
Deep‐eutectic solvents
Heterogeneous systems
Aqueous buffer with water‐immiscible organic solvent or ionic liquids
Pure substrate as emulsion or solid
Micellar systems

In addition to enabling substrate solubilization, using organic solvents can also alter an enzyme's selectivity. A study on the transesterification of octylhydroquinone (a natural product analog) with *n*‐butanol revealed that *Pseudomonas cepacia* lipase switches its regioselectivity. While the *meta*‐ester was favored fivefold in cyclohexane, the *ortho*‐ester was favored twofold in acetonitrile.^[^ ^33^ ^]^ For many lipases and proteases, the chemoselectivity, i.e., the preference for a hydroxyl group relative to an amino group for acylation in a given substrate, is strongly dependent on the solvent.^[^ ^34^ ^]^

Finally, the stereoselectivity can also be influenced and sometimes even reversed by solvents. The transesterification of the medicinally relevant compound methyl‐3‐hydroxy‐2‐phenylpropionate with propanol in different solvents with α‐chymotrypsin revealed a preference for the *S*‐enantiomer in cyclohexane and a preference for the *R*‐enantiomer in acetone.^[^ ^35^ ^]^ While enzymatic selectivity can also be fine‐tuned by protein engineering, these examples highlight opportunities for liquid phase engineering, which have been proven in single‐step biocatalysis and have not yet been transferred to in vitro biosynthetic cascades.

**Figure 2:**
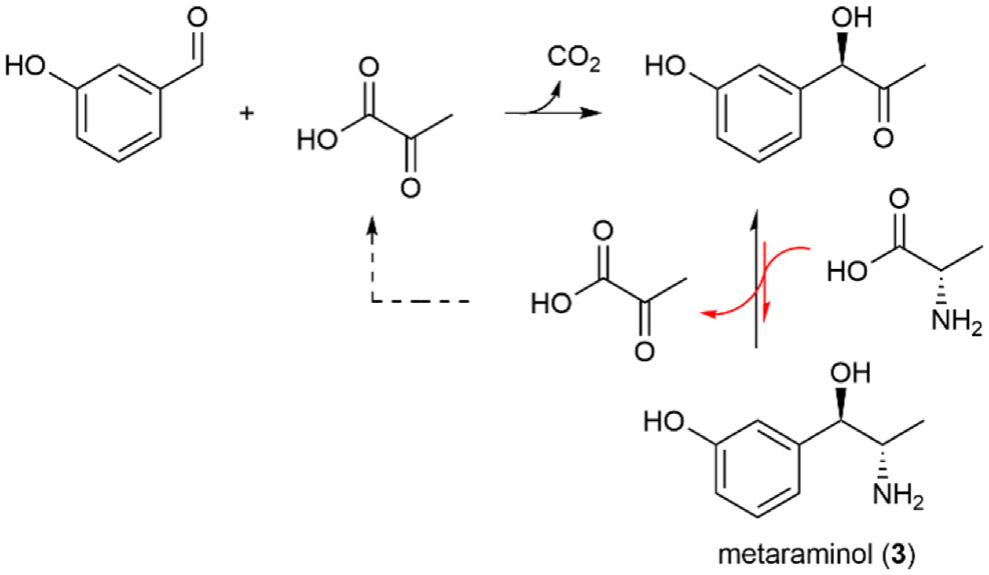
Enzymatic cascade toward metaraminol (**3**). The carboligation of 3‐hydroxybenzaldehyde and pyruvic acid is catalyzed by a thiamine diphosphate‐dependent pyruvate decarboxylase. Subsequently, a transaminase converts the intermediate (*R*)‐3‐hydroxy‐phenylacetylcarbinol into **3**. In situ liquid–liquid extraction of this compound shifts the transamination reaction toward the product side.

Apart from substrate solubility or enzyme selectivity, unfavorable reaction equilibria can also be addressed by reaction engineering. This is achieved either by integrating an irreversible reaction step that consumes a by‐product,^[^ ^36^ ^]^ or by continuous removal of the cascade end product.^[^ ^37^ ^]^ The latter approach, which is often referred to as extractive biocatalysis or in situ product removal (ISPR), also reduces the chance of product inhibition. Moreover, it minimizes potential product losses, which might arise from evaporation or cross‐interaction with other cascade components. Among the different ISPR techniques,^[^ ^38^ ^]^ the extraction into another phase (e.g., a water‐immiscible organic solvent) and the immobilization via adsorption onto water‐insoluble carriers are likely most convenient for in vitro biosynthesis. Through comparison of the product's physicochemical properties (log*P* and p*K*_a_ value, boiling point, etc.) with those of the other reactants, the appropriate ISPR technique can be selected. The preparation of the pharmaceutical drug metaraminol (**3**) from 3‐hydroxybenzaldehyde and pyruvate in a two‐step enzymatic cascade with l‐alanine as amine donor is a showcase for the use of ISPR, which helped to shift the equilibrium of the transamination reaction toward the product side (**Figure** **2**).^[^ ^37^ ^]^

**Figure 3:**
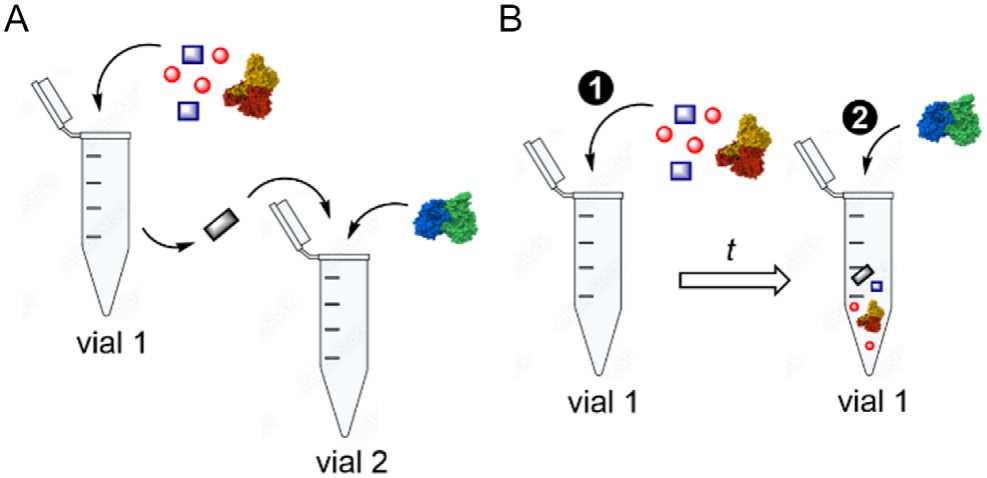
Operation modes for a two‐enzyme cascade. The reactions can be separated in space with an intermediary extraction step A) or in time B). The simultaneous operation mode where all reaction components are added at the same time, is not displayed.

In the case of incompatibilities within a cascade, e.g., between reactants and enzymes, the separation of certain reactions might become necessary. This separation can be accomplished either in time or in space (**Figure** **3**). A separation in space mimics the compartmentalization occurring in cellular systems. It means the abandonment of the one‐pot reaction concept and usually requires the intermediary extraction of one or more reaction intermediates, as illustrated in the production of **1**.^[^ ^11^ ^]^ This approach is also very useful to adjust the reaction conditions of the individual enzymatic reactions.^[^ ^39^ ^]^ A separation in time is even easier to implement. Instead of adding all cascade components to the reaction vessel simultaneously, some enzymes (or reactants) are withheld in the first instance and then supplemented in a timely fashion. In this way, not only can the inhibition of early reaction steps be prevented, but it is also possible to alleviate problems resulting from low enzyme stabilities or selectivities. Dirkmann et al. reported that the enzymatic conversion of acetate into patchoulol was only possible under the given buffer conditions, when the terminal pathway enzyme, a terpene synthase with low stability, was added 24 h after the cascade had been started.^[^ ^40^ ^]^ The enzymatic production of **3**, which is displayed in Figure 2, presumably also requires a timely separation of the two reaction steps, as the transaminase might be capable of accepting 3‐hydroxy‐benzaldehyde as an alternative substrate.^[^ ^41^ ^]^

It is important to stress that sequential operation modes for enzymatic cascades give the researcher a plethora of opportunities for improvements. In the production of the cyclic dinucleotide 2′,3′‐cGAMP, a biosynthetic pathway was reconstructed with enzymes from different organisms, including a polyphosphate kinase for ATP recycling from a thermophilic organism. Sequential addition of reactants and temperature adjustments resulted in an overall conversion of 57% of the substrates toward the product.^[^ ^42^ ^]^ The synthesis of cyclic dinucleotides also highlights an advantage of in vitro biosynthesis for exploiting enzyme promiscuity. Similar to precursor‐directed biosynthesis in cellular systems,^[^ ^10^ ^]^ derivatives of natural products can be obtained by using substrate analogs. In the case of 2′,3′‐cGAMP nine derivatives were obtained by using nucleotide derivatives as substrates.^[^ ^43^ ^]^

**Figure 4:**
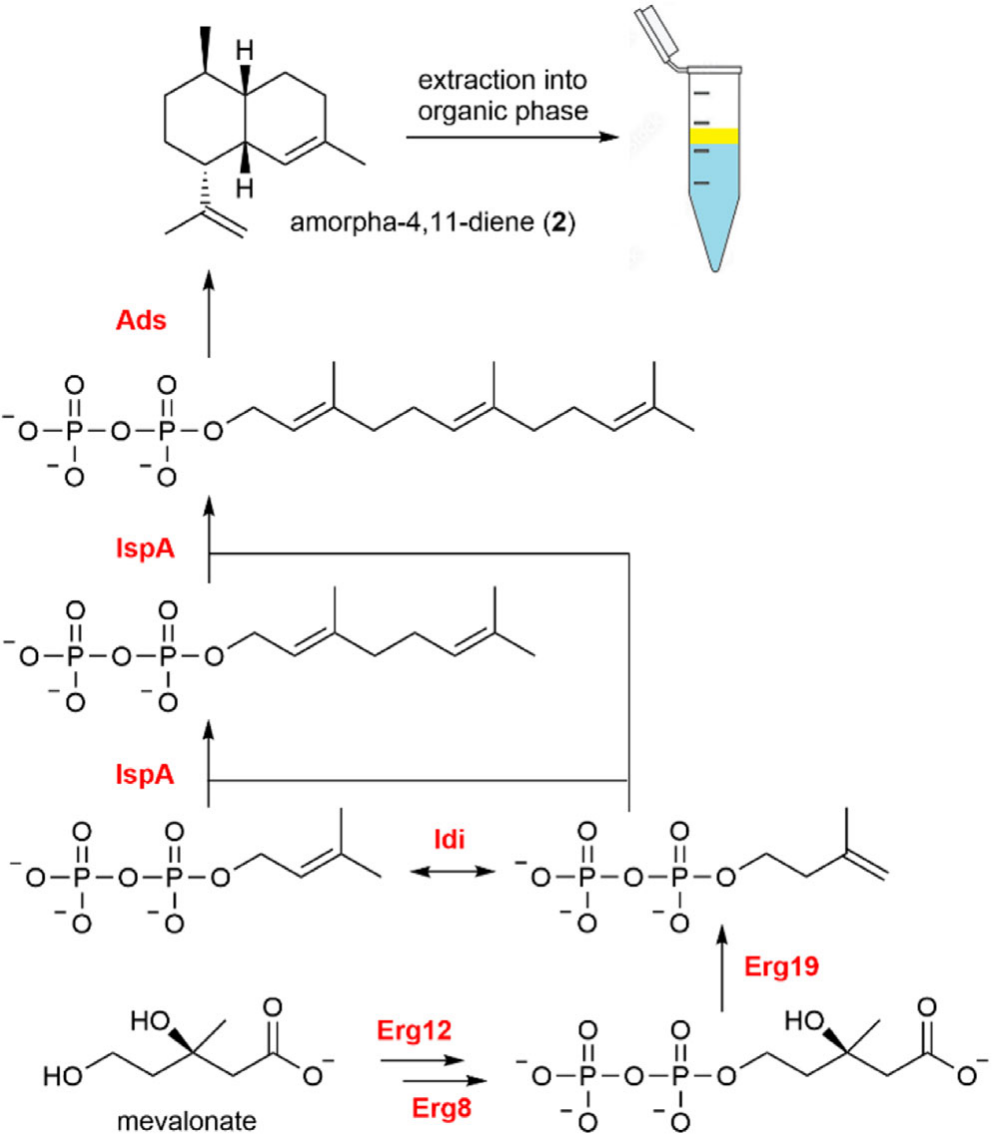
Enzymatic in vitro synthesis of amorpha‐4,11‐diene (**2**). The seven‐step cascade was reconstituted in a single reaction vial under two‐phase reaction conditions. The local optimum enzymatic activity ratio of the six involved enzymes was determined in a combinatorial approach designed with a factorial orthogonal array and response surface technology. Enzyme abbreviations: Erg12, mevalonate kinase; Erg8, phosphomevalonate kinase; Erg19, diphosphomevalonate decarboxylase; Idi, isopentenyl pyrophosphate isomerase; IspA, farnesyl pyrophosphate (FPP) synthase; Ads, amorpha‐4,11‐diene synthase.

The balancing of enzyme concentrations is another proven concept to improve the flux under in vitro conditions. This approach was successfully applied to the in vitro biosynthesis of the already mentioned patchoulol and explains, in part, the comparatively high yield of this cascade (Table 1).^[^ ^40^ ^]^ Still, many researchers rely on titration experiments for adjusting enzyme ratios in biosynthetic cascades, though statistical methods are becoming increasingly popular. The study by Chen et al. on the enzymatic synthesis of **2** underlines the enormous potential of this approach.^[^ ^16^ ^]^ The researchers not only identified the local optimum activity ratio of the six involved enzymes, but their model also allowed them to spot the critical accumulation of a biosynthetic intermediate, which negatively affected the yield of **2**. After solving this issue, which involved the fine‐tuning of enzymatic activity by buffer optimization, a complete conversion of the substrate mevalonate was achieved (**Figure** **4**).^[^ ^16^ ^]^

Mevalonate was also used as a substrate in an ATP‐regenerating artificial cascade, which was optimized by an algorithm‐based approach.^[^ ^44^ ^]^ The productivity of mevalonate phosphate was doubled by adjusting enzyme and substrate concentrations.

In vitro biosynthesis typically requires the integration of cofactor regeneration reactions to account for the lack of cellular metabolism. ATP supply was identified as one rate‐limiting factor in the in vitro biosynthesis of farnesyl pyrophosphate (FPP) by a combined model‐ and experiment‐based approach.^[^ ^45^ ^]^ These examples reiterate the importance of the fine‐tuning of the reaction environment to achieve high enzymatic rates, which in turn lead to higher productivity.

In conclusion, in vitro biosynthesis can be significantly improved by reaction engineering concepts established for single‐step biocatalysis by optimizing the liquid phase composition both in terms of enzyme ratios and reactant concentrations, as well as using media other than plain aqueous buffer solutions.

### 2.3 Computational Strategies

The study of the complex network of factors that regulates enzymatic activity at the molecular level paves the way for developing strategies to overcome the challenges associated with in vitro biosynthesis. Here, we focus on biomolecular simulations, which allow us to investigate complex biocatalytic systems to understand experimental observations and to deliver predictions and engineering strategies for experimental validation. We note that approaches such as biological networks, genome‐scale metabolic models, steady state and dynamic models, design‐build‐test‐learn cycles, and big data have been recently reviewed elsewhere^[^ ^27^ ^,^ ^46^ ^]^ and are out of the scope of this article.

Numerous computational studies have been reported addressing enzymes from different points of view, from engineering of the substrate specificity^[^ ^47^ ^]^ to solvent effects,^[^ ^48^, ^49^ ^–^ ^50^ ^]^ confinement,^[^ ^51^ ^]^ allosteric effects,^[^ ^52^ ^]^ and reaction mechanisms,^[^ ^53^ ^]^ to name a few. Given their complexity, it is not surprising that biosynthetic cascades have been less studied compared to one‐step enzymatic syntheses. On the other hand, the wealth of powerful computational techniques, which are frequently applied to single‐reaction processes, can also be exploited for the more demanding investigation of biosynthetic cascades. For these complex systems, additional aspects have to be considered, such as undesirable side reactions that may reduce the availability of the substrate for the next step in the cascade and the different stability/solubility patterns of the enzymes in the reaction media.

In this section, we focus on some of the main challenges for biomolecular simulations of biosynthetic systems by discussing selected examples and the application of computational tools to address these challenges.

#### 2.3.1 Predicting the Structure of the Enzymes Involved in the Biosynthetic Pathway

Atomic coordinates are needed as a starting point for most biomolecular simulations. If available, these coordinates are obtained from structures experimentally determined using techniques such as crystallography or NMR spectroscopy. However, when experimental structures are not available or their quality is poor, modeling offers an alternative. Homology modeling relies on two main observations: 1) the structure of a protein is determined by its amino acid sequence, and 2) during evolution, the structure is much more conserved than the sequence. This means that the unknown structure of a protein can be extrapolated using the known 3D structures of one or more proteins with similar sequences as templates. Useful homology models can be generated by employing templates with as little as 30% sequence identity with the protein to be modeled. In addition to sequence identity, the resolution of the template also contributes to the quality of the model. A good resolution is needed for good models. Several web servers and tools exist to build homology models, e.g., SWISS‐MODEL,^[^ ^54^ ^]^ Modeller,^[^ ^55^ ^]^ and Robetta.^[^ ^56^ ^]^ In recent years, various machine learning (ML) tools have been developed for predicting the structures of proteins.^[^ ^57^, ^58^ ^–^ ^59^ ^]^ AlphaFold2^[^ ^59^ ^]^ and AlphaFold3^[^ ^60^ ^]^ are perhaps the best‐known ML tools, with the latter also finding applications for predicting the 3D structure of protein complexes.

**Figure 5:**
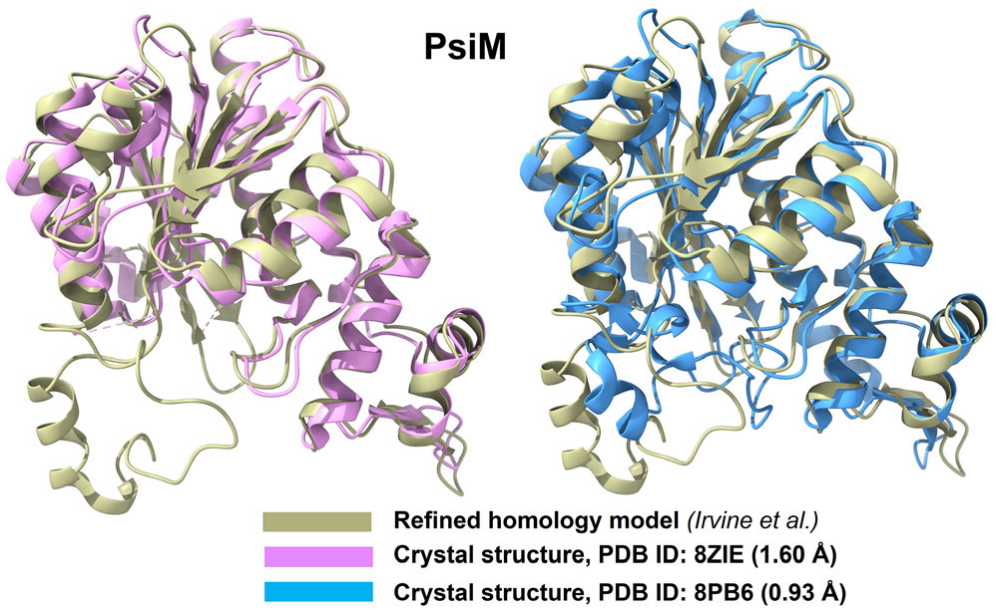
Overlay of the homology model of PsiM (olive) reported by Irvine et al.^[^ ^61^ ^]^ and the crystal structure of PsiM in its apo form (pink, PDB ID: 8ZIE).^[^ ^67^ ^]^ The overlay of the same homology model with the crystal structure of PsiM, obtained in complex with SAH and baeocystin (blue, SAH and baeocystin are not shown, PDB ID: 8PB6),^[^ ^66^ ^]^ is also displayed. As expected, less overlap is found at the highly flexible loop regions. The figures, shown in a new cartoon representation, were created using Chimera X.^[^ ^137^ ^]^

An illustrative example for in silico structure prediction related to biosynthetic cascades is the characterization of the psilocybin biosynthesis enzymes.^[^ ^61^ ^]^ Previous studies by the Hoffmeister group had demonstrated that the enzymatic pathway to this psychoactive natural product involves four enzymes, namely the phosphatidylserine decarboxylase‐like PsiD, the cytochrome P450‐like monooxygenase PsiH, the putative phosphotransferase PsiK, and the methyltransferase PsiM.^[^ ^62^ ^]^ Irvine et al.^[^ ^61^ ^]^ generated homology models of the four enzymes using a combination of techniques: SWISS‐MODEL^[^ ^63^ ^]^ and Robetta^[^ ^56^ ^]^ for identifying suitable templates and generating the initial structures, and Modeller version 10.0 for subsequent structural refinement.^[^ ^64^ ^]^ Not only was the resulting model of PsiM consistent with a previously reported homology model,^[^ ^65^ ^]^ but it also showed good agreement with experimental structures reported later (**Figure** **5**).^[^ ^66^ ^,^ ^67^ ^]^

**Figure 6:**
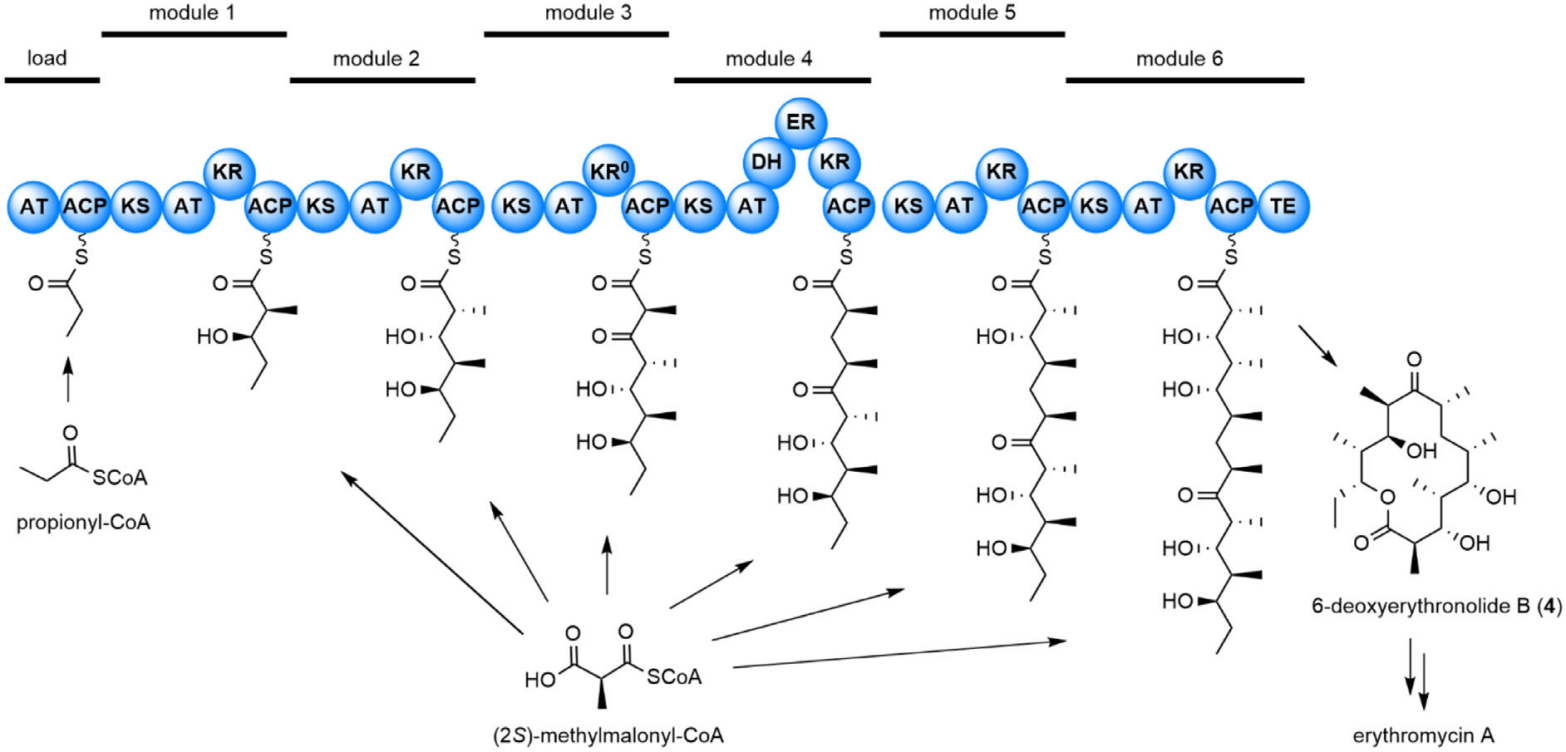
Modular organization of the DEBS enzyme complex and assembly‐line biosynthesis of 6‐deoxyerythronolide B (**4**). The loading module is primed with propionyl‐CoA, while modules 1 to 6 utilize (2*S*)‐methylmalonyl‐CoA as an extender unit. Domain notation: AT, acyl transferase; ACP, acyl carrier protein; KS, *β*‐ketoacyl synthase; KR, ketoreductase; DH, dehydratase; ER, enoyl reductase; TE, thioesterase.

Sundermann et al. delivered an engineering strategy for 6‐deoxyerythronolide B synthase (DEBS).^[^ ^47^ ^]^ This modularly organized polyketide synthase (PKS) enzyme complex assembles the macrolide scaffold of the antibiotic erythromycin by a specific set of covalently linked catalytic domains. In DEBS, the acyltransferase (AT) domains of modules 1–6 are specific towards (2*S*)‐methylmalonyl‐CoA (**Figure** **6**). The protein engineering efforts focused on the AT of the sixth DEBS module (DEBS‐AT6), for which no experimental structure was available. The AT3 and AT5 domains of DEBS share sequence identities with AT6 above 40%. Therefore, AT3 and AT5 were employed as templates for building the homology model of AT6, using the tool I‐TASSER.^[^ ^68^ ^]^ The validated homology model of AT6 was the basis of further computational studies enabling the rational engineering of DEBS by targeted mutagenesis.^[^ ^47^ ^]^

#### 2.3.2 Predicting the Binding of Substrates

The structural characterization of substrate‐enzyme complexes is key for tuning the substrate selectivity of enzymes via protein engineering and for developing novel substrates. Docking approaches allow predicting the binding region and poses of the substrate in the enzyme. Unlike other scenarios, such as drug design studies, in many enzymes, the binding region of the substrate is known, as it will comprise the enzyme's active site. If allosteric effects play a role and the allosteric site is unknown, blind docking strategies must be employed. Unlike in local docking, where the binding of the ligand focuses on a predetermined protein region or on specific regions, in blind docking, the exploration covers all the accessible surface of the protein. These approaches can be implemented in combination with rigid docking, where protein and ligand are considered rigid, or with flexible docking, where geometric restraints can be applied to atoms, but the overall flexibility of the system is still accounted for. The main challenges of docking approaches involve the effective sampling of the conformational space of the protein–ligand system (here, enzyme‐substrate) and the accurate scoring of the resulting complexes. In other words, multiple alternatives (poses) to where and how the ligand binds to the protein must be generated, trying to cover as much as possible of the desired conformational space and taking into account the degrees of freedom of the ligand itself. Stochastic, deterministic, or systematic approaches are used for searching the conformational space.^[^ ^69^ ^]^ Depending on the system, several tens of thousands to hundreds of thousands of poses may be generated within a single docking calculation. These resulting poses must be ranked in a manner that is reliable and fast at the same time, since the landmark of docking algorithms is precisely their computational efficiency. Generally speaking, scoring functions^[^ ^70^ ^]^ can be classified in order of decreasing accuracy but increased computational efficiency as force field‐based, empirical potentials, and knowledge‐based potentials, which can benefit from the growing development of ML approaches. Mixed approaches combine different types of search algorithms and scoring functions. Several docking tools are available, such as Autodock Vina^[^ ^71^ ^]^ and SwissDock,^[^ ^72^ ^,^ ^73^ ^]^ Gold,^[^ ^74^, ^75^ ^–^ ^76^ ^]^ Glide,^[^ ^77^, ^78^ ^–^ ^79^ ^]^ and Rosetta Dock (ROSIE),^[^ ^80^ ^]^ among many others.

Docking approaches were also applied to the two case studies previously discussed in this Computational strategies section.^[^ ^47^ ^,^ ^61^ ^]^ Once the homology model was generated and protonation states of titrable residues assigned, the DEBS‐AT6 model was used as a starting point for rigid docking of the native substrate, using the subsequent biomolecular simulations.^[^ ^47^ ^]^ For the in silico characterization of the psilocybin enzymes,^[^ ^61^ ^]^ the substrates l‐tryptophan, tryptamine, 4‐hydroxytryptamine, as well as norbaeocystin and baeocystin were docked into PsiD, PsiH, PsiK, and PsiM, respectively. In addition, methylated derivatives of psilocybin and 4‐hydroxytryptamine, as well as a dimethylated derivative of tryptamine, were docked into PsiM. Flexible docking was performed using AutoDock Vina.^[^ ^71^ ^]^ The resulting complexes were analyzed to propose key residues for binding.

Importantly, the accuracy of the scoring function very much influences the reliability of docking predictions. The error range of the score estimations should be taken into account when discussing the scoring results. For instance, the predicted binding affinities of different docking poses frequently lie within the 1–2 kcal mol^−1^ range, which is within the error range of the best scoring functions. Thus, it cannot be assumed that one structure will be preferred among others, based on these scores. It is not uncommon that the best‐scored structure resulting from docking calculations is not the actual one, as established using experimental data.^[^ ^81^ ^,^ ^82^ ^]^ A good practice is to analyze several of the complexes rather than overestimating the discerning power of scoring functions. When possible, the assessment of how well these predicted structures support known experimental data should be incorporated into the analysis of the results of docking calculations. Proof‐of‐concept calculations are also important. There, the same docking protocol is used to predict similar protein–ligand complexes for which experimental structures are available.

As the conformational search space is much larger, blind docking approaches may deliver less reliable results with respect to local docking. Re‐scoring a reduced set of protein‐ligand complexes with more accurate methods can also improve the reliability of docking predictions. It is often advisable to follow consensus strategies in which several docking tools are employed. Another strategy is the subsequent refinement of the results using molecular dynamics (MD) simulations or, in some cases, free energy estimations.

#### 2.3.3 Following the Dynamics of the Biocatalytic System in Explicit Media

MD simulations allow following the time‐dependent motion of the particles composing the biomolecular system. These particles (atoms in full atomistic simulations) are treated as points with a certain mass and a fixed charge (in non‐polarizable models), using Newton dynamics. Typically, the conformational energy of the system is calculated using molecular mechanics (MM) force fields. A force field is defined by the set of equations and parameters designed to reproduce the molecular geometry and selected properties of the simulation system. Typically, in MM force fields, these equations are potential energy functions that comprise bonded and non‐bonded terms accounting for bond stretching, angle bending, and torsion, as well as for electrostatic and van der Waals interactions. The theory and practice of MD simulations, including classical and enhanced sampling approaches, have been reviewed elsewhere.^[^ ^83^, ^84^ ^–^ ^85^ ^]^

Although more computationally demanding than fast docking approaches, MD simulations can be used to predict the binding of a ligand to a protein by placing the ligand far away from the target, allowing the system to evolve in time until binding occurs and no substantial changes are observed. This procedure is subsequently repeated with different initial positions of the unbound ligand. In MD simulations, the solvent can be considered using explicit solvation models where solvent molecules are explicitly treated.^[^ ^86^ ^]^ Implicit models are an alternative, where solvent effects are described using a continuum medium.^[^ ^87^, ^88^ ^–^ ^89^ ^]^ By neglecting the degrees of freedom of the solvent, the latter approach is computationally more efficient than explicit solvation. On the other hand, an important disadvantage of implicit solvation is that explicit interactions involving solvent molecules are not taken into account, affecting the quality of the results.

MD simulations are also an important step for physics‐based free energy calculations approaches.^[^ ^90^, ^91^, ^92^ ^–^ ^93^ ^]^ Free energy estimations, which have been reviewed elsewhere,^[^ ^90^ ^,^ ^91^ ^]^ can be used for predicting the substrate's binding affinity, for computational mutagenesis and protein engineering studies, among other applications. Several suites of programs are available for performing MD simulations. Diverse releases of Nanoscale Molecular Dynamics (NAMD),^[^ ^94^ ^]^ Groningen Machine for Chemical Simulations (GROMACS),^[^ ^95^ ^,^ ^96^ ^]^ Assisted Model Building with Energy Refinement (AMBER),^[^ ^97^ ^,^ ^98^ ^]^ and Chemistry at Harvard Macromolecular Mechanics (CHARMM)^[^ ^99^ ^,^ ^100^ ^]^ are among the software solutions most widely employed.

In the psilocybin study, the homology models of the four enzymes were refined using MD simulations, prior to the docking calculations.^[^ ^61^ ^]^ The authors used GROMACS^[^ ^95^ ^]^ version 2020.4 with the CHARMM27 force field.^[^ ^101^ ^]^ MD simulations, with the NAMD 2.7 program and the CHARMM22 force field, were also employed for the refinement of the homology and docking models of DEBS‐AT6^[^ ^47^ ^]^ and for examining enzyme–substrate interactions. The analysis of the MD simulations indicated that a largely conserved residue (Val295_DEBS‐AT6_) acted as a ‘gatekeeper’ preventing the incorporation of building blocks larger than the native substrate. On this basis, a DEBS‐AT6 variant featuring the V295A mutation was engineered to exhibit substrate promiscuity. Furthermore, in a follow‐up study, MD simulations were carried out to successfully predict the incorporation of several substrates into the DEBS‐AT6_V295A_ variant and to investigate the effects of substrate flexibility on polyketide biosynthesis.^[^ ^102^ ^]^

In the field of enzymatic cascades, the biosynthesis of monensin involves a PKS that incorporates ethyl‐ or methylmalonyl‐CoA. The corresponding AT domain at the PKS's fifth module (AT5_mon_) was investigated. Using Modeller v. 9.10,^[^ ^103^ ^]^ Bravo‐Rodriguez et al. built homology models of AT5_mon_ to study the mechanisms of substrate recognition.^[^ ^104^, ^105^ ^]^ The best homology models were used for MD simulations (with NAMD2.9^[^ ^106^ ^]^ and the CHARMM22^[^ ^107^ ^]^ force field) in the absence and presence of several substrates. In addition, using Free Energy Perturbation theory,^[^ ^108^ ^,^ ^109^ ^]^ relative Gibbs energy differences (ΔΔ*G*) were calculated to evaluate the affinity of the active site of AT5_mon_ for the substrates. The simulations delivered a molecular understanding of the relaxed specificity in AT5_mon_ and insights into the activation of AT5_mon_ for the nucleophilic attack on the substrate, as well as predictions concerning the incorporation of synthetic malonic acid building blocks.

MD simulations find many applications beyond the refinement of structural models of enzymes and enzyme‐substrate complexes, the prediction of protein–ligand interactions, and free energy calculations. MD simulations can nowadays reach the microseconds to milliseconds time scale (or even longer depending on the system). Thus, MD can be employed to follow the enzyme's conformational changes upon allosteric activation and/or substrate binding, as well as to study the effect of the liquid environment on the enzyme and enzyme‐substrate interactions. Importantly, MD simulations are the computational method of choice for the detailed study of the dynamic behavior of the liquid media at the molecular level, which is one of the most complex factors influencing the behavior of biocatalytic systems.

#### 2.3.4 Studying Reactivity

Typically, MD simulations in biocatalysis are based on the classical MM formalism, where the electronic structure of the atoms is not considered and the bond distances between atoms are fixed around equilibrium distances. This means that the breaking and the formation of chemical bonds (as in chemical reactions) cannot be simulated using classical MM force fields. Quantum Mechanics (QM), on the other hand, explicitly accounts for the electronic structure of the atoms, thus allowing the study of chemical reactions. However, the full QM treatment of biocatalytic systems is, for most cases, not viable. Although reactive force fields have been developed to address reactivity at the force field level,^[^ ^110^, ^111^, ^112^ ^–^ ^113^ ^]^ hybrid multiscale QM/MM approaches remain the method of choice for the study of chemical reactions in biocatalytic systems.^[^ ^114^, ^115^ ^–^ ^116^ ^]^ In QM/MM methods, the simulation system is artificially partitioned into a region to be treated quantum mechanically (for instance, the active site of the enzyme, substrate, and any other relevant molecule in the vicinity) and the rest of the biomolecule and its environment, which are treated at the MM level. This way, the simulation becomes viable by employing the most computationally demanding electronic structure method only for the region for which electronic effects (such as reactivity) must be accounted, while the rest of the system is treated with the less accurate but more computationally efficient MM force field.

In the PKS studies discussed here,^[^ ^47^ ^,^ ^105^ ^]^ QM/MM optimizations were used for calculating, at a higher level of theory, the interactions of the substrates at the active site of the AT domains (DEBS‐AT6 and AT5_mon_) and to validate the force field parameters of the substrates. The ChemShell^[^ ^117^ ^]^ v3.2 platform was employed for the QM/MM calculations, where the QM region (the substrate) was treated at the density functional level and the rest of the system with the CHARMM force field.

#### 2.3.5 Studying Biosynthetic Cascades

Beyond single enzymatic reactions, computational techniques can be exploited for the investigation of biosynthetic cascades. These techniques also allow studying side reactions and enzyme stability in different media and environmental conditions as well as the characterization of substrates and reactants. In many cases, by focusing on the individual steps of the biosynthesis, the needed molecular insights are acquired, and substrate, media, and protein engineering strategies can be devised. A first systematic approach to the relevant steps is always advised, even if specific, more complex features inherent to the cascade must be included later in the model.

The fact that in a biosynthetic cascade, the reactions are linked together and often take place in complex reaction environments adds an extra layer of difficulty to the simulations. Compartmentalization is another challenge. For very large systems, coarse‐grained (CG) modeling is an alternative. In CG approaches,^[^ ^118^ ^,^ ^119^ ^]^ the degrees of freedom of the simulation system are reduced by grouping several heavy atoms and their associated hydrogen atoms into beads that represent the properties of the atoms’ cluster. This allows reducing the total number of particles and degrees of freedom in the system, and thus simulating larger biomolecular systems and longer time scales. Due to its versatility, the Martini force field is widely used for CG biomolecular simulations.^[^ ^120^ ^,^ ^121^ ^]^

Due to the approximate representation of the biomolecular system in which groups of atoms are replaced by beads, the improved computational efficiency of coarse‐graining comes at the cost of missing fine structural details. Back‐mapping approaches,^[^ ^122^ ^]^ which include a minimization step, rely on transforming the CG resolution back to atomistic resolution to recover the atomistic structure necessary to analyze molecular interactions after a CG MD simulation.

CG hybrid approaches have been implemented with QM/MM methods among them.^[^ ^123^, ^124^ ^–^ ^125^ ^]^ QM/MM/CG approaches allow coarse‐graining a part of the system while still preserving the rigorous quantum mechanical treatment of the region that should be treated at the electronic structure level, as well as the MM description of the remaining full atomistic region. CG approaches are very useful for studying compartmentalized enzymes, where the simulation setup can comprise millions of particles. For instance, the Southamerican Initiative for a Rapid and Accurate Hamiltonian CG force field^[^ ^126^ ^]^ was successfully applied to study the role of confinement in the catalytic properties of an allosterically regulated protease.^[^ ^51^ ^]^

Understanding and tuning the effect of the liquid media on biosynthetic cascades is perhaps one of the most challenging tasks, since the same cosolvent or additive may exert a different action, depending on the system. In these complex systems, biomolecular simulations provide very valuable insights. For instance, MD simulations allow rigorously monitoring of the MD of solvent–solvent, solvent–substrate, solvent–protein interactions, and analyzing their effect on the whole catalytic system. Other parameters, such as temperature and pressure can also be adjusted in MDs to account for experimental conditions. Free energy estimations can indicate which media favor the incorporation of the substrate at the active site or deliver the free energy associated with transferring the biocatalytic system from one liquid medium to another. QM/MM studies shed light on the specific involvement of the media in the reaction mechanism.

As shown with AlphaFold, ML‐based tools complement biomolecular simulations. The interplay between ML‐ and physics‐based approaches will grow even stronger with the growing development of ML applications. For instance, ML potentials have been developed for MD simulations^[^ ^127^ ^,^ ^128^ ^]^ and ML tools have been developed for protein engineering.^[^ ^129^, ^130^, ^131^ ^–^ ^132^ ^]^ These data‐based approaches are complemented by physics‐based methods, which in turn deliver phenomenological, molecular, and atomistic foundations for the rational engineering of biocatalytic systems.

## 3 Summary and Outlook

Natural product biosynthesis is based upon enzyme‐catalyzed reaction cascades. While the reconstruction of biosynthetic reaction sequences under in vitro conditions is already well established, substrate turnover and product yields are often low in such systems without optimization of the reaction conditions.

In this review, we have presented various concepts from the areas of reaction engineering and biomolecular simulation that are useful for the design of enzyme‐catalyzed reactions and that enable an improvement of the cascade performance.

Still, the optimization of biosynthetic cascades is a challenging task that, at the moment, is typically conducted empirically. The fundamentals to identify the best conditions for biosynthetic cascades are often not rationalized in detail, which is also owing to the complexity of these systems. Biosynthetic cascades comprise multiple interwoven reactions that take place in liquids. In contrast to gas‐phase reactions, reactions in liquids are to a large extent determined by interactions between reactants, products, solvents, and the other ingredients of the liquid system. These interactions may influence both the performance of enzymes and the thermodynamic activity of reactants and products. They may alter solubilities, structural properties (e.g., in the case of enzymes), transition‐state barriers, and thus reaction kinetics or may even change reaction paths. These manifold interactions depend on the physical properties of the involved chemical species, such as size, 3D structure, polarity, hydrogen‐bond donating/accepting abilities, or charges. Since these properties are interconnected, they cannot be easily interpreted as independent factors contributing to an observed effect.

In recent years, several attempts have been made to address the design of liquid media for simple enzymatic reactions. Advances in computational chemistry enable the representation of solvents either implicitly, e.g., as a dielectric continuum, or as explicit species, and provide molecular insights into complex solvent effects in (bio)catalysis.^[^ ^133^ ^,^ ^134^ ^]^ These works mainly addressed the influence of solvents on single‐step reactions performed at infinite dilution of the reactants in that solvent. Although the effect of solvents on reactants at finite and hence technically relevant concentrations can be accounted for by biomolecular and thermodynamic modeling,^[^ ^135^ ^,^ ^136^ ^]^ complex systems, such as biosynthetic cascades, or the simultaneous influence of the liquid reaction medium on both enzyme stability and reactant thermodynamic activity have not been addressed yet.

The development of physics‐based models for biosynthetic cascades is still hindered by the scarcity of experimental data reporting the molecular interactions in these systems. Such experimental data, complemented by in silico studies, are crucial to understand the origin of the non‐linear and non‐additive interplay between enzymes, reactants, and solvent. Advances in data‐based approaches, in particular hybrid methods combining physical models with ML algorithms, are best positioned to enable liquid phase design for in vitro biosynthesis. These hybrid approaches can draw upon the strength of mechanistic understanding of physiochemical theory, as well as tackle the many interactions in complex liquid systems by data‐based methodologies, which also benefit from experimental data.

## Conflict of Interest

The authors declare no conflict of interest.
